# Developing a novel framework for non-technical skills learning strategies for undergraduates: A systematic review

**DOI:** 10.1016/j.amsu.2018.10.005

**Published:** 2018-10-09

**Authors:** Marios Nicolaides, Luca Cardillo, Iakovos Theodoulou, John Hanrahan, Georgios Tsoulfas, Thanos Athanasiou, Apostolos Papalois, Michail Sideris

**Affiliations:** a Barts and the London School of Medicine and Dentistry, Queen Mary University of London, 4 Newark St, Whitechapel, London, E1 2AT, United Kingdom; b Faculty of Life Sciences and Medicine, King's College London, Great Maze Pond, London, SE1 9RT, United Kingdom; c Department of Surgery, Aristotle University of Thessaloniki, Thessaloniki, 54124, Greece; d Imperial College London, Faculty of Medicine, Department of Surgery and Cancer, South Kensington Campus, London, SW7 2AZ, UK; e Experimental Research Centre ELPEN, 95 Marathonos Avenue, 19009, Pikermi, Greece; f Obstetrics and Gynecology, London Deanery, United Kingdom

**Keywords:** Non-technical skills, Communication skills, Undergraduate medical education, Framework, Learning

## Abstract

**Objectives:**

There is substantial lack of guidance when it comes to the implementation of non-technical skills (NTS) in undergraduate medical education. This review aimed to identify and critically evaluate published literature on learning strategies for NTS in undergraduate medical education and to derive a training framework targeted towards standardizing future training interventions.

**Methods:**

A systematic review of the MEDLINE database was performed using a prospective protocol following PRISMA guidelines. Studies evaluating undergraduate medical students exposed to NTS interventions, which measured subjective or objective outcomes in selected attributes, were included.

**Results:**

Initial systematic search yielded a total of 5079 articles, out of which 68 fulfilled the inclusion criteria. A total of 24 NTS were identified, with communication skills being the most commonly reported skill evaluated (n = 37). A variety of educational tools were used (n = 32), noteworthy being the use of simulated patients. Great heterogeneity was also observed in measured outcomes and methods of assessment. A ‘triad of outcomes’ in NTS training was devised (knowledge, skill performance and attitude towards skills) and used for classification of all reported outcomes. Extracted data were used to design a non-technical skill training framework.

**Conclusions:**

The existing literature describes a plethora of NTS interventions in undergraduate medical education, with varied outcomes and assessments. We hereby propose the ‘NTS Training Framework’, in an attempt to coordinate future research and catalyze the identification of an ideal NTS course structure to form tomorrow's physicians.

## Highlights

• Initial systematic search yielded a total of 5079 articles, out of which 68 fulfilled the inclusion criteria.
• A total of 24 non-technical skills (NTS) were identified, with communication skills being the most commonly reported skill.
• A ‘triad of outcomes’ in NTS training was devised (knowledge, skill performance and attitude towards skills).
• We propose a unified framework for NTS training, with the objective of guiding future research.

## 1 Introduction

In an era of globalized medicine and increased public pressure for high-quality care, the need to form medical professionals with greater adaptability to social environments is ever-growing. Whilst knowledge and technical skills remain indispensable pillars of medical education, non-technical skills (NTS) training has attracted considerable attention in recent decades [1], aiming to contribute to a more holistic model of medical education. NTS can be defined as a mélange of ‘soft skills’ allowing doctors to self-evolve as part of a ‘learning organization’ capable of adapting in volatile environments [[2], [3], [4]]. Increasing use of the term “soft skills” pertains to a paradigm shift from the medical profession's traditional notions of internalized norms and implicit standards towards a culture of audits, transparency and self-surveillance.

Inspired by its original application in the aviation sector and air safety, NTS training implementation has expanded to many multidisciplinary fields, including healthcare, to prevent adverse outcome related to human factors errors [5,6]. NTS training aspires to resolve healthcare failures precipitated by errors often conceived at the organizational level. For instance, narratives surrounding failures such as the Mid-Staffordshire scandal, revealed conditions that often lay the groundwork for errors, favouring quick fixes and ‘blame games’ over learning and transparency within teams [7,8]. Such systemic ‘defects’ have been the focus of numerous high-impact reports such as ‘To Err is Human’ and ‘A promise to learn–a commitment to act’ [7,9]. Yet preventable harm continues to occur, exacerbating both patient suffering and healthcare costs [10]. Even at the undergraduate level, lack of practice, anxiety and reduced confidence are all factors contributing to students' under performance when interacting with patients or operating within multidisciplinary environments [11]. These shortcomings may be traceable to the lack of a universal NTS training framework, highlighting the need for a unified and focused training approach part of medical school curricula [[12], [13], [14]].

NTS training efforts are the product of two main driving forces. The first, arising in the 1970s and based on the concept of ‘dehumanization’, sustains that medical students become progressively ‘estranged’ and detached from patients throughout their training, leading to compromised patient care - a theory also supported by more recent studies [15,16]. The second relates to the potential of NTS to influence clinical outcomes independently of technical skills [17]. Indeed, studies suggest that NTS, such as effective doctor-patient communication, ensure better health outcomes, patient safety, satisfaction and compliance [18], and decreased patient distress [19]. Equally, doctors benefit from a gratifying work environment and reduced malpractice lawsuits [20,21].

Despite the widely recognized advantages of comprehensive NTS training, research surrounding its implementation remains elusive. Whilst many studies evaluate NTS interventions, at present there are no clear guidelines for implementing NTS learning strategies. With this in mind, we performed a systematic review (SR) to identify and critically evaluate published literature on learning strategies for NTS in undergraduate medical education. Additionally, we outline comprehensive NTS intervention outcomes and derive a NTS training framework targeted for standardizing future training interventions.

## 2 Methods

We performed a SR following PRISMA guidelines to identify studies evaluating NTS interventions in undergraduate medical education. Studies were hand-searched to find additional papers not included in the initial search.

### 2.1 Registration

This systematic review has been registered with Research Registry (registration code: reviewregistry608).

### 2.2 Assessment of methodological quality of the systematic review (AMSTAR 2)

We completed the AMSTAR 2 checklist to assess the quality of our methodology [22].

### 2.3 Search strategy

The search strategy focused on pooling studies published on the MEDLINE database, including targeted ‘non-technical skills’ intervention strategies. We utilized an extensive list of keywords obtained from MeSH terms pertaining to all qualities and skills-other than technical proficiency-believed to play a role in the development of future doctors; a complete list of all included keywords can be viewed in Appendix 1. This list was compiled following detailed review of key precedent studies [14,23] as well as the General Medical Council's (GMC) “Outcomes for graduates” guidelines, outlining key competencies expected of newly qualified doctors [24].

### 2.4 Selection criteria

**Fig. 1:**
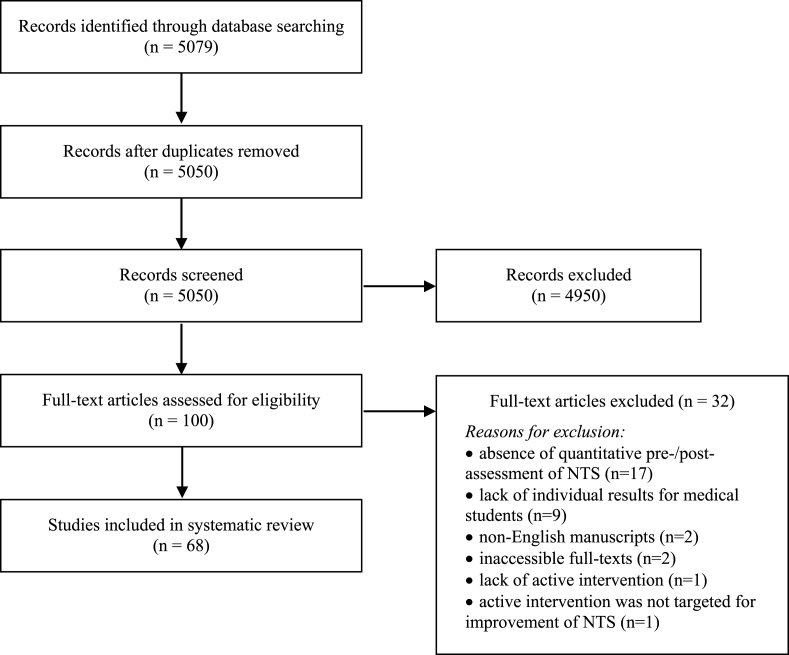
PRISMA flow diagram [25].

As part of the SR protocol we agreed to a “Population, Intervention, Comparison, Outcome – PICO” strategy (Fig. 1). Inclusion criteria limited selected studies to only those exposing primarily undergraduate medical students or mixed medical with other healthcare students (P, population) to any non-technical skills training approach incorporated within the curriculum of, or offered by, a Medical Institution or any other provider (I, intervention). Included studies were furtherly limited to those measuring perceived or actual changes in attributes such as skills, knowledge or attitudes in randomized or quasi-experimental study designs (C, comparison). Outcomes sought were the objective or perceived improvement in participants' attributes. In borderline cases, study inclusion was made by default. Exclusively qualitative studies, reporting students’ perceptions towards intervention (NTS module) were not considered. Such studies did not seem to affect any of our primary results. We also excluded studies not reporting baseline performance of the subjects, and therefore unable to comment on the impact of the intervention (performance improvement).

### 2.5 Data extraction

To maximize the homogeneity of extracted information from shortlisted studies, we used a pilot, prospectively designed worksheet, structured around the PICO headings. Extracted fields were based on the subheadings as indicated in Table 1. Whenever study location was not provided, the presumed location was the corresponding authors’ affiliation country. A third reviewer (I.T.) was involved in the full-text articles assessment and resolved any disagreement between the two reviewers (M.N., L.C.), cross-checking and confirming the validity of extracted data. Any further disagreement was discussed and dissolved by the senior authors (M.S., A.P.). The final extraction sheet was standardized to provide refined results amenable to more accurate qualitative analysis and subsequent synthesis of results.

**Table 1:** PICO data extraction fields.

PICO Criteria	Extracted fields
General	Aim, Year and Location of study
Population	Intervention/Control group sizes
	Demographic details
	Tutor/Facilitator background
	Recruitment method
	Year of study
Intervention/Control	Non-technical skill assessed
	Educational tool used
	Use of simulated patients
	Duration of intervention
Outcomes	Attribute assessed
	Tool of assessment
	Conclusion of study
Other	Study limitations

## 3 Results

### 3.1 Selected studies

The initial systematic search yielded a total of 5079 records from MEDLINE. Following removal of duplicates, 5050 records were screened against our inclusion criteria. A total of 100 studies were selected from primary screening, and the full-text articles retrieved for eligibility assessment. A total of 68 studies were eligible for inclusion in our SR. (Fig. 1).

### 3.2 Study characteristics

**Fig. 2:**
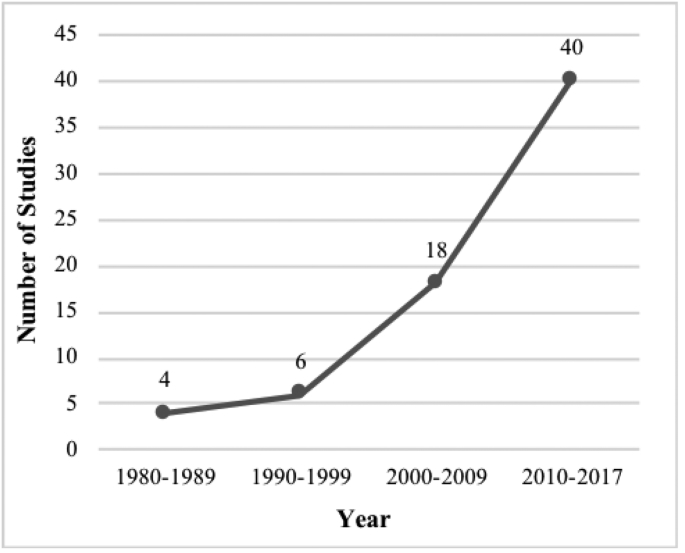
Studies assessing NTS interventions, 1980-Present.

Fig. 2 shows a remarkable increase in the number of studies available per year since 1980, with a striking increase of 122% between periods 2000–2009 and 2010–2017. Most studies were featured in the Americas (n = 25) [[26], [27], [28], [29], [30], [31], [32], [33], [34], [35], [36], [37], [38], [39], [40], [41], [42], [43], [44], [45], [46], [47], [48], [49], [50]], followed closely by Europe (n = 24) [[51], [52], [53], [54], [55], [56], [57], [58], [59], [60], [61], [62], [63], [64], [65], [66], [67], [68], [69], [70], [71], [72], [73], [74]]. Specifically, the majority were carried out in the United States (US) (n = 23) [[26], [27], [28], [29], [30], [31], [32], [33], [34], [35], [36], [37], [38], [39], [40], [41], [42], [43], [44], [45], [46], [47], [48]], whereas the United Kingdom (UK) ranked second with 10 studies [[51], [52], [53], [54], [55], [56], [57], [58], [59], [60]]. Of 68 included studies, 48 reported both qualitative and quantitative measures, whilst the remainder were solely quantitative.

### 3.3 Study population

Study population was homogeneous, with medical students forming the main participant group in selected studies. 11 studies also involved other healthcare students [28,32,34,39,46,47,56,57,62,66,75], with nursing students being the most represented group (n = 7) [28,39,47,56,57,66,75]. As summarized in Table 3, the total sample size ranged from 7 to 373 medical students, with an average of 111 ± 87.1 per study. Two papers were not considered for such average given that participant number was not provided. Indeed, tutors' number and profession were provided by only 24 and 22 studies respectively; both of these factors were considered while designing the ‘NTS Training Framework’.

Variability was observed in the disclosure of further participant demographic information, specifically concerning first language, gender and mean age. Only 24 papers specified whether students had previous experience in NTS learning [26,28,32,35,39,41,44,45,52,55,61,63,64,[67], [68], [69],[74], [75], [76], [77], [78], [79], [80], [81]].

### 3.4 Intervention

After exploring the learning outcomes of the included studies, we defined an NTS intervention as ‘*any teaching strategy aiming to improve an individual's performance, knowledge and attitude towards a non-technical skill*’. A total of 24 discrete NTS interventions were identified, with communication and empathy skills being featured a total of 37 and 9 times respectively (Table 3). We described studies as either long or short, based on intervention duration greater than 40 hours (active time) or 12 weeks (total time). When duration was defined in days, we assumed one working day equals to 8 hours. Considering these parameters, we computed an average study duration of 33 ± 94.1 hours and described 25 studies as long (Table 3). Of the 68 studies, 15 did not specify the exact intervention time and were therefore omitted from the aforementioned calculations [30,32,33,38,42,43,50,53,54,57,72,77,82,83]. Courses were either (a) implemented in the medical schools' core curricula and made compulsory to all attending students (n = 46) or (b) optional to internal medical students (n = 22).

As anticipated, a variety of educational tools were utilized (32 in total). Simulated patients (SP) were used in 31 studies and of those, 16 (52%) utilized SP feedback either as an educational tool or as a method of assessment. Didactic lectures, video-assisted learning and role play were recurrently integrated in the courses generating a combination of both traditional (didactic lectures) and non-traditional methods of teaching in most NTS courses. Table 2 summarizes the 16 most commonly used educational tools with corresponding frequency, whilst the remaining (n = 16) were only utilized once each.

**Table 2:** Educational tools used with corresponding frequency.

Educational Tool	Number of times used
Didactic lecture	25
Role play	25
Video assisted learning	25
Simulated Patients	21
Workshop	21
Feedback	20
Other	16
Group discussion	11
Clinical placement	9
Seminar	7
Case Based Learning	4
E-learning	4
Audio record feedback	3
Practical	2
Problem Based Learning	2
tOSCE	2
Tutorial	2

**Table 3:** Summary of interventions in eligible studies.

Author	Non-technical skill	Length of Intervention	Simulated Patient (SP) used	Sample size (Medical students)	Year groups	
					Pre-clinical (1–2)	Clinical (3–6)
Aboumatar et al. [44]	Communication skills Teamwork skills	Short	No	120	✓	
Alroy et al. [79]	Interpersonal skills	Short	No	n/a		✓
Ayuob et al. [84]	Communication skills	Short	No	293		✓
Betson et al. [85]	Breaking bad news	Short	No	160		✓
Blatt & Greenberg [36]	Teaching skills	Short	No	103		✓
	Communication skills					
	Feedback-giving skills					
Bonnaud-Antignac et al. [72]	Breaking bad news	Long	Yes	108		✓
Braniff et al. [51]	Communication skills	Long	No	240		✓
	Teaching skills					
	Understanding the work environment					
	Teamwork skills					
	Learning skills					
Buczacki et al. [52]	Interprofessional collaboration	Short	No	331		✓
Cämmerer et al. [70]	Communication skills	Long	Yes	84	✓	
Carpenter [56]	Interprofessional collaboration	Short	No	23		✓
Carter et al. [31]	Cross-cultural training	Short	No	196		✓
Chun & Lee [86]	Debating skills	Long	No	45	✓	
Dixon-Woods et al. [60]	Communication skills	Long	No	173	✓	
Doherty et al. [65]	Communication skills	Short	Yes	127		✓
Efstathiou & Walker [57]	Communication skills	Short	No	14		✓
Engerer et al. [69]	Communication skills	Short	Yes	34		✓
Engler et al. [43]	Communication skills	Long	No	46	✓	
Erickson et al. [26]	Communication skills	Short	Yes	118		✓
	Teamwork skills					
Fadlon & Pessah [87]	Interviewing skills	Long	No	56	✓	
	Empathy skills					
	Communication skills					
Fernández-Olano et al. [62]	Empathy skills	Short	No	137	✓	
Fletcher et al. [58]	Emotional Intelligence	Long	No	86		✓
Forsgren et al. [61]	Communication skills	Short	Yes	69		✓
Franco et al. [64]	Communication skills	Short	Yes	69		✓
Hagemann [68]	Situation awareness	Short	No	77		✓
	Teamwork skills					
	Task management					
	Decision-making					
Hagemeier et al. [39]	Communication skills	Short	Yes	73	✓	
Haidet et al. [29]	Communication skills	Short	No	34		✓
Hammer & Rian [48]	Presentation skills	Short	No	7	✓	✓
Harlak et al. [88]	Communication skills	Long	No	59	✓	
Heiman et al. [33]	Presentation skills	Long	Yes	132	✓	
Hess et al. [46]	Communication skills	Long	Yes	67	✓	
Hobgood et al. [47]	Teamwork skills	Short	No	235		✓
Hobgood et al. [47]	Breaking bad news	Short	Yes	138		✓
Joekes et al. [54]	Communication skills	Long	Yes	82	✓	
Karnieli-Miller et al. [89]	Interpersonal communication skills	Short	Yes	19	✓	
	Humor					
Knox and Bouchier [53]	Communication skills	Long	Yes	n/a	✓	
Konopasek et al. [27]	Communication skills	Short	Yes	90		✓
	Feedback-giving skills					
Koponen et al. [73]	Communication skills	Long	Yes	129	✓	
Kushner et al. [30]	Empathy skills	Short	Yes	127	✓	
	Communication skills					
Lanken et al. [34]	Communication skills	Long	No	370	✓	✓
Lau et al. [81]	Communication skills	Short	No	160	✓	
Lie et al. [42]	Interpreter interaction skills	Short	No	304	✓	
Lim et al. [83]	Empathy skills	Long	No	77		✓
Lim et al. [77]	Empathy skills	Short	No	72		✓
LoSasso et al. [35]	Empathy skills	Short	No	70		✓
Loureiro et al. [63]	Communication skills	Long	No	115		✓
Ludwig et al. [38]	Teamwork skills	Short	Yes	373		✓
Lukman et al. [82]	Communication skills	Long	Yes	189	✓	
Martino et al. [45]	Brief Motivational Interviewing	Short	Yes	45		✓
Mauksch et al. [32]	Communication skills	Long	No	22		✓
Moorhead & Winefield [90]	Empathy skills	Short	No	63		✓
Ozcan et al. [75]	Empathy skills	Short	No	143	✓	
Poole & Sanson-Fisher [78]	Empathy skills	Short	No	45		✓
Rees & Sheard [55]	Communication skills	Long	Yes	216	✓	
Robertson et al. [28]	Teamwork skills	Short	Yes	104		✓
Rosen et al. [40]	Cross-cultural training	Short	Yes	32		✓
Saab et al. [91]	Communication skills	Short	No	75	✓	
Schildmann et al. [66]	Breaking bad news	Short	Yes	23		✓
Shapiro et al. [50]	Communication skills	Long	Yes	79	✓	
Simmenroth-Nayda et al. [67]	Communication skills	Long	Yes	32		✓
Tiuraniemi et al. [74]	Communication skills	Short	No	126		✓
Todisco et al. [76]	Interviewing skills	Long	Yes	60	✓	
Tsai et al. [92]	Interviewing skills	Short	Yes	27		✓
Usherwood [59]	Interviewing skills	Short	No	44		✓
von Lengerke & Kursch [71]	Communication skills	Short	Yes	267	✓	
Wiese et al. [41]	Presentation skills	Short	No	62		✓
Yeung et al. [49]	Teaching skills	Long	No	18	✓	
	Communication skills					
Yu et al. [93]	Non-verbal communication skills	Short	No	82	✓	
Zgheib et al. [80]	Communication skills	Long	No	102	✓	
	Professionalism					
	Personal Development					

### 3.5 Learning outcomes

**Fig. 3:**
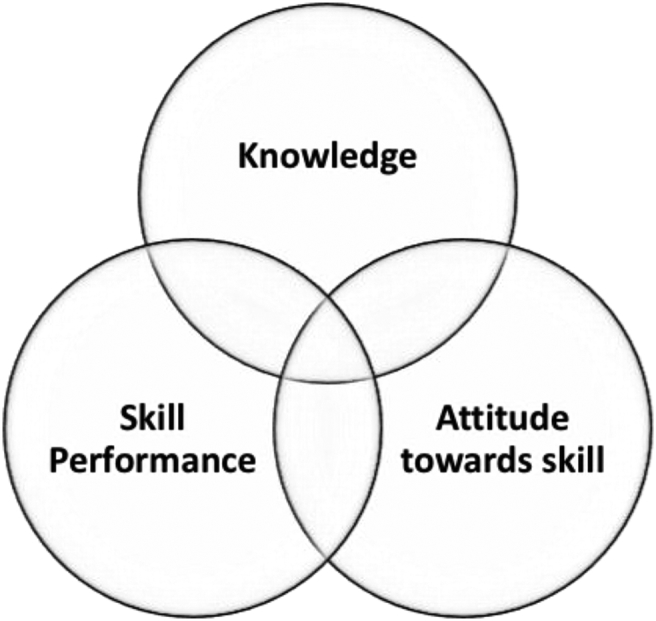
Triad of outcomes in a NTS intervention.

We noted an extensive variation in learning outcomes amongst selected studies, possibly owing to the broad pool of courses, each one assessing different NTS. Inspired by the apparent gap in literature concerning universal NTS outcomes, we devised a ‘triad of outcomes’. This approach encapsulates a step-wise model by which students potentially acquire new NTS, with 1) knowledge of the NTS providing the initial context and scaffolding to the subsequent 2) performance of the NTS, followed by 3) self-reflection on the learning accomplished. Indeed, our ‘triad’ represents three potentially overlapping, albeit distinct, steps which both medical students and educators should focus upon when reflecting on their performance and designing interventions, respectively (Fig. 3). An overlap between assessed outcomes is inevitable as some skills impact various improvement areas.

We used the following definitions for classification of the reported triad of outcomes:

1. *Knowledge*: theoretical understanding of the principles surrounding a specific NTS.
2. *Skill performance*: quantifiable adeptness in a specific NTS.
3. *Attitude towards skill*: subjective perception about the usefulness and purpose of a specific NTS.

By qualitatively synthesizing (Table 4) the reported outcomes in accordance with the aforementioned ‘improvement areas’, we were able to deduce some descriptive statistics. For example, *skill performance* was encountered most frequently, amounting to 60 times, while *attitude towards skill* and *knowledge* appeared on 31 and 16 occasions, respectively.

**Table 4:** Summary of study outcomes and assessment.

Author	Outcome			Assessment					
	Skill Performance	Knowledge	Attitude towards skills	Assessment Method 1	Assessment Method 2	Assessment Method 3	Assessment Method 4	Validated method	Results
Aboumatar et al. [44]	✓	✓		Likert scale questionnaire	System thinking scale			✓	+
Alroy et al. [79]	✓			Rating scale				✓	+
Ayuob et al. [84]	✓	✓		Multiple-choice questionnaire	Checklist based assessment				+
Betson et al. [85]	✓			Likert scale questionnaire					+
Blatt & Greenberg [36]	✓	✓	✓	Learners' rating	Likert scale questionnaire	Interaction analysis data			+
Bonnaud-Antignac et al. [72]	✓	✓		Likert scale questionnaire	6-point protocol			✓	+
Braniff et al. [51]	✓			Likert scale questionnaire					+
Buczacki et al. [52]	✓			Likert scale questionnaire					+
Cämmerer et al. [70]	✓			Likert scale questionnaire					+
Carpenter [56]		✓	✓	Likert scale questionnaire					+
Carter et al. [31]			✓	CABS^a^					+
Chun & Lee [86]	✓			Debate Competence Scale				✓	+
Dixon-Woods et al. [60]		✓	✓	Likert scale questionnaire					+
Doherty et al. [65]	✓		✓	Likert scale questionnaire					+
Efstathiou & Walker [57]	✓		✓	Likert scale questionnaire					+
Engerer et al. [69]	✓			Likert scale questionnaire					+
Engler et al. [43]	✓			CSIC^b^				✓	+
Erickson et al. [26]	✓		✓	SCS^c^	JSAPNC^d^	ATHCT^e^		✓	+
Fadlon & Pessah [87]		✓	✓	Likert scale questionnaire	Focus groups				+
Fernández-Olano et al. [62]	✓			JSPE^f^				✓	+
Fletcher et al. [58]	✓			Bar-On EQ-i				✓	+
Forsgren et al. [61]	✓		✓	Likert scale questionnaire	Checklist-based assessment				+
Franco et al. [64]	✓	✓		Multiple-choice questionnaire	CCPQC-CC^g^	CSAS^h^	Likert scale questionnaire	✓	+
Hagemann [68]	✓		✓	ANTS Observation System^i^	Likert scale questionnaire			✓	+
Hagemeier et al. [39]	✓			Likert scale questionnaire					+
Haidet et al. [29]	✓		✓	Likert scale questionnaire	MAAS^j^	PPOS^k^		✓	+
Hammer & Rian [48]	✓		✓	Likert scale questionnaire					+
Harlak et al. [88]	✓		✓	CSAS^h^	Empathic Tendency Scale			✓	**-**
Heiman et al. [33]	✓			Checklist based assessment					+
Hess et al. [46]	✓			Common Ground Rating Scale	OSCE^l^			✓	+
Hobgood et al. [47]	✓	✓	✓	Teamwork attitudes instrument	Teamwork knowledge test			✓	+
Hobgood et al. [47]	✓		✓	Likert scale questionnaire					+
Joekes et al. [54]	✓		✓	Doctor-Patient (DP) Scale	Likert scale questionnaire	Interview Rating Scale	OSCE^l^	✓	+
Karnieli-Miller et al. [89]	✓		✓	Patient–Practitioner Orientation Scale	Lavie Scale	RCS^m^		✓	+
Knox and Bouchier [53]		✓		Modified essay question					+
Konopasek et al. [27]	✓		✓	Likert scale questionnaire					+
Koponen et al. [73]			✓	CSAS^h^				✓	+
Kushner et al. [30]	✓		✓	Likert scale questionnaire					+
Lanken et al. [34]	✓	✓	✓	Likert scale questionnaire	JSPE^f^			✓	+
Lau et al. [81]	✓			Likert scale questionnaire					+
Lie et al. [42]		✓		Multiple-choice questionnaire					+
Lim et al. [83]	✓		✓	JSPE^f^	OSCE^l^			✓	+
Lim et al. [77]	✓		✓	Behaviour Change Counselling Index				✓	**-**
LoSasso et al. [35]	✓		✓	JSPE^f^				✓	+
Loureiro et al. [63]	✓		✓	STAI^n^	Interpersonal Behaviour Survey	CSAS^h^		✓	**-**
Ludwig et al. [38]	✓			Likert scale questionnaire					+
Lukman et al. [82]	✓	✓	✓	General Information questionnaire	ICI^o^	CSAM^p^	CSVA^q^	✓	+
Martino et al. [45]	✓	✓		Likert scale questionnaire	Multiple-choice questionnaire				+
Mauksch et al. [32]	✓			Likert scale questionnaire					+
Moorhead & Winefield [90]	✓			Empathy Rating Scale					**-**
Ozcan et al. [75]	✓		✓	ECSS^r^	Empatic Tendency Scale			✓	+
Poole & Sanson-Fisher [78]	✓			Accurate Empathy Scale				✓	+
Rees & Sheard [55]			✓	CSAS^h^				✓	**-**
Robertson et al. [28]	✓	✓	✓	Teamwork knowledge test	CHIRP Scale^s^	Team skills checklist video rating		✓	+
Rosen et al. [40]	✓	✓	✓	Likert scale questionnaire					+
Saab et al. [91]	✓			Rating scale					+
Schildmann et al. [66]	✓			Likert scale questionnaire					+
Shapiro et al. [50]	✓		✓	SAICQ^t^	SPIR^u^	ISRS^v^		✓	**-**
Simmenroth-Nayda et al. [67]	✓			CCOG^w^				✓	+
Tiuraniemi et al. [74]	✓			Likert scale questionnaire	Global self-appraisal of competency				+
Todisco et al. [76]	✓			Likert scale questionnaire	Videotaped interview rating scale				+
Tsai et al. [92]	✓			Videotaped interview rating scale					+
Usherwood [59]	✓			Likert scale questionnaire					+
von Lengerke & Kursch [71]	✓			Likert scale questionnaire					+
Wiese et al. [41]	✓			Videotaped interview rating scale					+
Yeung et al. [49]	✓			Likert scale questionnaire					+
Yu et al. [93]	✓			METT^x^	SETT^y^				+
Zgheib et al. [80]	✓			TPS^z^				✓	+
**TOTAL**	**60**	**16**	**31**					**30**	

**Abbreviations**: **a**: Cultural Attitudes and Beliefs Scale, **b**: Carkhuff's Standard Index of Communication, **c**: Self-efficacy in Communication Scale, **d**: Jefferson Scale of Attitudes toward Physician-Nurse Collaboration, **e**: Attitudes toward Health Care Team, **f**: Jefferson Scale of Physician Empathy, **g**:Clinical Communication and Professionalism Questionnaire of Capability – Communication Competencies, **h**: Communication Skills Attitude Scale, **i**: Anaesthesia Non-Technical Skills Observation System, **j**: Mindful Attention Awareness Scale, **k**: Patient Practitioner Orientation Scale, **l**: Objective Structured Clinical Examination, **m**: Relational Communication Scale **n**: Speilberger's State-Trait Anxiety Inventory, **o**: Interpersonal Communication Inventory, **p**: Communication Skills Attitude Measure **q**: Communication Skills Video Assessment, **r**: Empathic Communication Skill Scale, **s**: Collaborative Healthcare Interdisciplinary Relationship Planning, **t**: Self-Assessment of Interpersonal Competence Questionnaire, **u**: The Staff-Patient Interaction Rating Scale, **v**: Interpersonal Skills Rating, Scale, **w**: Calgary-Cambridge Observation-Guide, **x**: Micro Expression Training Tool, **y**: Subtle Expression Training Tool, **z**: Team Performance Scale.

### 3.6 Assessment

Our analysis yielded a total of 49 individual methods of assessment (Table 4), which we classified as either objective or subjective. Objective methods of assessment reflect the actual performance (n = 23), whilst subjective methods of assessment reflect the perceived competence of the participant in the relevant skill (n = 26). Of the 68 studies, 53 included a subjective method, either on its own or in conjunction with an objective one. In 30 studies (44%), a validated method of assessment was used, nevertheless many of the remaining studies failed to report whether methods used were validated.

Overall study results after NTS intervention were classified as having either a positive or negative impact on the study population, based upon the provided data and conclusions drawn by the authors (Table 4). Only six papers reported negative findings, with a decline in measured outcomes following the intervention enacted. This may suggest the presence of publication bias towards positive results.

### 3.7 Limitations of the included studies

Included studies reported several limitations, the most common being the absence of a control group (n = 19). This was followed by a small sample size (n = 16) and the application of the NTS intervention in question to a single institution only (n = 12). Few studies reported on the long-term retention of the intervention undertaken, and this was mentioned as a limitation to 10 studies. Further to this, based on AMSTAR 2 checklist the methodological quality is low, which can be attributed to significant heterogeneity in the studies included.

## 4 Discussion

The modern shift of patient care to the biopsychosocial approach demands a coincident change in medical education, particularly in non-technical skills teaching [94,95]. This review has compiled substantial evidence supporting early exposure of undergraduate medical students to NTS training. Evaluation of included studies has been complicated by the heterogeneity of reported participant and educator demographics, interventions, outcomes, and their assessment. This underscores the need to unify NTS teaching implementation and reporting. To this effect, we propose a singular NTS teaching framework.

### 4.1 Parameter 1: Participants and educators

NTS training was compulsory in the majority of studies included, and we suggest it be a mandatory element of medical school curricula (Table 5). Results showed participants were mostly in the clinical years of their studies, however it is unclear whether introduction of NTS training in clinical versus pre-clinical years has particular advantages. Few studies specified students’ socio-demographics and previous experience in NTS, complicating appraisal of the important link between such and NTS acquisition [[96], [97], [98]]. This hinders our efforts to optimize future course designs, particularly if neglected student minorities exist undetected by the current literature. Similarly, the optimum number of students per intervention cannot be established reliably due to: 1) variation in group size dependent on the educational tool used and 2) variation in the student to tutor ratio. Acknowledging these ambiguities, our framework helps overcome them by standardizing the reporting of results such that future studies can be compared more accurately (Table 5).

**Table 5:** Parameters of NTS training framework.

Parameter		Suggestions
Course	Participants	• Recruitment: immediate mandatory attendance by implementation of course into the core curriculum. • Year group: pre-clinical (years 1–2), clinical (years 3–5) or both. • Demographic information: previous experience, first language, gender and mean age.
	Educators	• Training: tutors should be trained to standardize quality of course delivery. • Number: as per institution's availability.
	Intervention	• Identification of elements of NTS based on a previously validated skill taxonomy. • Educational tools: (a) combination of both traditional and non-traditional tools (b) mandatory use of a simulation tool (simulated patients, role play etc.). • Active intervention time: 20–30 hours. • Frequency: longitudinal course. Aid retention with “spiral” curriculum.
	Outcomes	• Participant's overall performance to be assessed in the following areas: (a) Knowledge (b) Skill performance (c) Attitude towards skill
Assessment	Nature	• Pre-/Post- intervention assessment • Control group
	Methods	• Use of established assessment methods: (a) Pre-existent, validated methods of assessment (b) Likert scale ratings of NTS subdivided into domains • Combination of subjective methods (attitudes) and objective methods (performance and knowledge).

Educator information is also poorly represented, with most studies failing to provide sufficient analyzable data. Given the pivotal role of educators in NTS training, we propose to alleviate any potential disparities in educators’ backgrounds with a uniform pre-course training (Table 5).

### 4.2 Parameter 2: Intervention

The vast number of different NTS intervened for in the analyzed studies reinforces our argument to unify training by defining a core set of NTS for the undergraduate curriculum. To accomplish this, we propose a ‘frequency-based’ approach, selecting the most common NTS and training strategies from the existing evidence base. This assumes educational demand has spurred coincidental development of relevant NTS study interventions. For example, the frequent use of communication skills interventions (37 times) may suggest recognition of the importance of this NTS and students' need for improvement in this domain. Indeed, the literature demonstrates a shift away from ‘classic’ areas of intervention previously incorporated in surgical, anesthetic and health education frameworks, towards skills such as empathy and breaking bad news [3,4,99]. Whilst this approach guarantees catering for the current educational needs, it's effectiveness is dependent upon continued monitoring of NTS learning.

Currently, the majority of reported interventions are limited to domain-specific training, focusing on singular aspects of the medical profession such as the operating room (OR) [3,4,100]. This observation highlights a future pathway for NTS training, where movement out of the OR and into surgical wards can be mirrored in other areas of the medical profession [101].

Present educational methods combine traditional and novel training tools. Simulation-Based Learning (SBL) has been implemented in a variety of medical disciplines [102,103], with recent evidence of its role in NTS training [[104], [105], [106], [107], [108], [109]]. Despite its sparse use in the selected studies, evaluating its time advantages and high fidelity, we consider SBL a fundamental part for all future NTS interventions [103,110,111]. Also, we appraise the increasing use of feedback from SP noted in our study. Though a recent review [112] was inconclusive regarding feedbacks’ effectiveness in aiding medical students, it has been suggested that feedback can positively affect communication skills when used in conjunction with other education tools, warranting further investigation [113]. Taking into consideration the above points, we encourage use of simulation in NTS training in conjunction with other widely-accepted traditional or non-traditional methods of teaching (Table 5).

To the best of our knowledge, no existing studies correlate NTS active intervention duration to participant outcomes. However, most authors support a longitudinal model, involving a course extending over weeks or months as opposed to singular timepoints [114,115]. Positive outcomes for participants are generally reported by studies, independently of intervention time or frequency. This may indicate that duration and frequency are not pivotal factors towards the success of NTS training. However, we exercise caution when drawing scheduling suggestions, as our calculation of study duration was based on assumptions (see results), whilst publication bias towards positively-skewed results may also exist. Nonetheless, due to the time constraints pre-existing in medical school curricula, we propose that the ideal NTS intervention duration should verge towards our average of 20–30 hours, distributed longitudinally.

Whilst most studies show NTS training leads to participant learning, retention of skills can degrade over time [43,78,116]. Maintenance of high quality NTS throughout a physician's career has the potential to improve clinical outcomes and care standards; hence, we argue it is vital to periodically revisit NTS throughout undergraduate education. This also addresses the natural decline in students' NTS, including empathy, experienced over the duration of medical school [[117], [118], [119]]. This can be achieved by scheduling regular NTS training over the academic years, building upon previous NTS taught, whilst increasing student proficiency through further, more complex exercises [120].

### 4.3 Parameter 3: Outcomes

The extensive variation in learning outcomes reported in the reviewed studies constituted a barrier to interpreting the relative impact of the NTS interventions on students. To simplify this, we developed a “triad of outcomes”, unifying measured outcomes in three areas: 1) knowledge, 2) skill performance, 3) attitude towards skill. Whilst this provides some basis for analysis, it is inherently limited in attempting to normalize outcomes disparate as the NTS they refer to. To eliminate this in future studies, we propose the ecumenical utilization of unified outcomes such as our “triad”, on the footsteps of the subdivision of NTS in tools such as the surgeons' NOTSS and anesthetists’ ANTS [2,3].

### 4.4 Parameter 4: Nature and method of assessment

The commonest rationale for study exclusion in this review was the lack of student assessment before and after the NTS intervention (Fig. 1). Also, many studies failed to utilize a control group not participating in any NTS intervention (n = 50). These limitations denote the inherent complexity of implementing a well-designed and controlled NTS intervention trial to a cohort of medical students. This is chiefly impeded by preexisting time constraints and the educational disadvantage of offering NTS training to only a cohort of students. Hence, variables such as students’ previous exposure to NTS training and to other curricular activities that may contribute to their NTS development cannot be compounded for. To obviate this shortcoming in future studies, we suggest the use of baseline and post-intervention assessments, which can also be “formative” assessments to students [121,122]. We also suggest the use of control groups, which can receive the NTS intervention subsequently, thus also permitting improved tutor to student ratios (Table 5).

Whilst many studies evaluated the same NTS, different assessment methods were often used, complicating the comparison of training effectiveness. To address this, we propose that future studies utilize unified assessment methods. A possible solution is to reaffirm assessment methods already commonly utilized, such as the *Communication Skills Attitude Scale* and the *Jefferson Scale of Physician Empathy* [123,124]. Amongst the reviewed studies these tools were the most frequently occurring, suggesting they may be easily extended to all future NTS intervention studies [4]. Importantly, these scales have been extensively validated, demonstrating good test-retest reliability and internal consistency, both in their English and non-English translations [88,[123], [124], [125], [126], [127], [128], [129], [130], [131]]. Likert scales were also utilized by a majority of the included studies. This suggests a second route to implement a common NTS assessment method, mimicking tools such as the NOTSS and the ANTS, grading NTS by Likert scales in key domains [2,3]. Whilst an attractive option, the success of this unifying method is dependent on reaching a consensus on the domains to be included. Moreover, subdivision of NTS into domains could allow assessment through feedback and self-evaluation, methods invaluable to capture the social skills involved in many NTS [4]. Applied to our proposed “triad” of outcomes, *attitude towards skill* could be best assessed via a subjective scale, such as a Likert scale or one of the validated tools aforementioned, whilst *skill performance* and *knowledge* could be measured via more objective tools, including multiple-choice questions, as exemplified by some included studies [42,45,64,84] (Table 5).

### 4.5 NTS in the transition from a personal to an organizational level

**Fig. 4:**
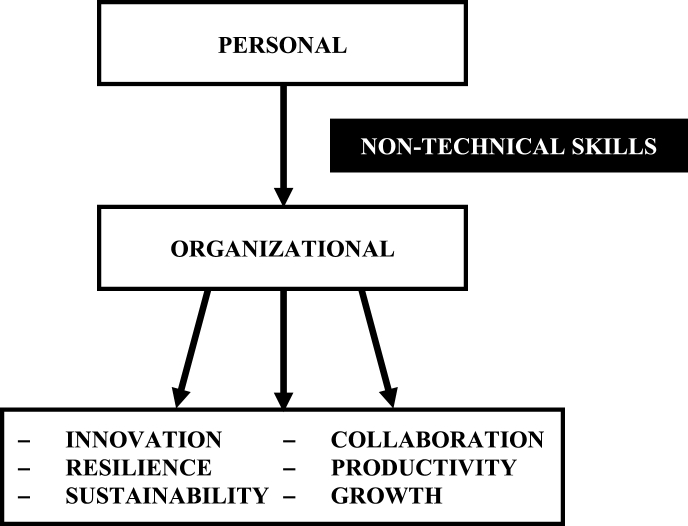
Transition from a personal to an organizational proficiency level depends upon competence in non-technical skills.

NTS should be viewed as a catalyst for improving personal competence and performance in multi-disciplinary teams and settings. Such personal gains lead to improved organizational performance and allow the individual to ‘mature’ and form a key prerequisite for organizational gains. We suggest that effective plurality can lead to innovation, resilience, sustainability, collaboration, productivity and growth (Fig. 4) – all key principles of high quality and cost-efficient care. On the basis of evidence from the compiled studies, and considering the damaging repercussions in the absence of such elements, we strongly support the early introduction of NTS training in undergraduate medical education.

### 4.6 Limitations

We have performed a systematic review of the literature on the MEDLINE database only. Albeit 68 articles were included for full text retrieval, this is a restriction of our study, and future work should include studies in other databases. Furthermore, the intrinsic diversity of NTS teaching studies included prejudiced the use of currently approved tools for appraisal of quality of evidence. Whilst we believe this would not have altered our conclusions, we recognize this as a limitation which should be addressed in further developments of our framework.

## 5 Conclusion

It is evident that integration of NTS training in undergraduate education continues to face challenges, illustrated in part by the huge increase in emerging studies but more importantly by the striking inconsistency between them. We propose a unified framework for NTS training, with the objective of guiding future research, facilitating comparison between interventions, and spurring the creation of a standardized NTS course. Although this review focuses solely on personal gains from the medical perspective, coordinated efforts to achieve similar gains in allied healthcare professionals are expected to trigger multiplier effects. Future studies will be required to elucidate the current theories on NTS teaching, in the endeavor to enhance the education of tomorrow's doctors.

## Provenance and peer review

Not commissioned, externally peer reviewed.

## Ethical approval

Not required.

## Sources of funding

Self funded.

## Author contribution

MN drafted the manuscript and is the lead author; parts of the manuscript have been drafted and edited by LC and IT. MN, LC, IT, JH performed data extraction and synthesis of evidence. MS, AP GT, TA are the senior authors with input in several steps of the authorship. MS, MN and AP conceived study, search strategy and data synthesis. All authors have approved final manuscript.

## Conflicts of interest

None declared.

## Trial registry number

NA.

## Research registration number

reviewregistry608.

## Guarantor

MS and AP are the guarantors of this work. MN is the lead author.

### Availability of data and material

The datasets supporting the conclusions of this article are included within the article (and its additional files). The raw data extraction from the reviewed studies can be shared by the corresponding author upon reasonable request.

## Appendix A Supplementary data

Appendix 1 Appendix 1

AMSTAR 2 AMSTAR 2
