# Supplementary material for: Developing a novel framework for non-technical skills learning strategies for undergraduates: A systematic review
Source: Ann Med Surg (Lond). 2018 Oct 9;36:29–40. doi: 10.1016/j.amsu.2018.10.005 (PMC6199815; doi:10.1016/j.amsu.2018.10.005)
Supplement: Appendix 1 [file mmc1.doc]

# Appendix 1

| **#** | **Query** | **Items found** |
| --- | --- | --- |
| **1** | ((Non-technical skills) OR Soft skills) OR Communication skills | 28348 |
| **2** | (((((((((((((((((((((((Situation awareness) OR Decision making) OR Teamwork skills) OR Leadership skills) OR Time management) OR Project management) OR Problem solving) OR Interpersonal skills) OR Positive attitude) OR Confidence) OR Work ethic) OR (Flexibility and adaptability)) OR Negotiation) OR Conflict resolution) OR Self-promoting) OR Net-working) OR Persuasion) OR Mentoring) OR Stress Management) OR Strategy Skills) OR Learning skills) OR Career development skills) OR Emotional Intelligence) OR Presentation skills | 1212488 |
| **3** | ((((Undergraduate) OR Medical education) OR Medical students) OR Undergraduate curriculum) OR Undergraduate curricula | 415926 |
| **4** | ((((((Teaching) OR Training) OR Learning) OR Assessment) OR Course) OR Seminar) OR Module | 3312052 |
| **5** | **#1 AND #2 AND #3 AND #4**  ((((((Non-technical skills) OR Soft skills) OR Communication skills)) AND ((((((((((((((((((((((((Situation awareness) OR Decision making) OR Teamwork skills) OR Leadership skills) OR Time management) OR Project management) OR Problem solving) OR Interpersonal skills) OR Positive attitude) OR Confidence) OR Work ethic) OR (Flexibility and adaptability)) OR Negotiation) OR Conflict resolution) OR Self-promoting) OR Net-working) OR Persuasion) OR Mentoring) OR Stress Management) OR Strategy Skills) OR Learning skills) OR Career development skills) OR Emotional Intelligence) OR Presentation skills)) AND (((((Undergraduate) OR Medical education) OR Medical students) OR Undergraduate curriculum) OR Undergraduate curricula)) AND (((((((Teaching) OR Training) OR Learning) OR Assessment) OR Course) OR Seminar) OR Module) | **5079** |
