# Supplementary figures and images for: Developing a novel framework for non-technical skills learning strategies for undergraduates: A systematic review
Source: Ann Med Surg (Lond). 2018 Oct 9;36:29–40. doi: 10.1016/j.amsu.2018.10.005 (PMC6199815; doi:10.1016/j.amsu.2018.10.005)

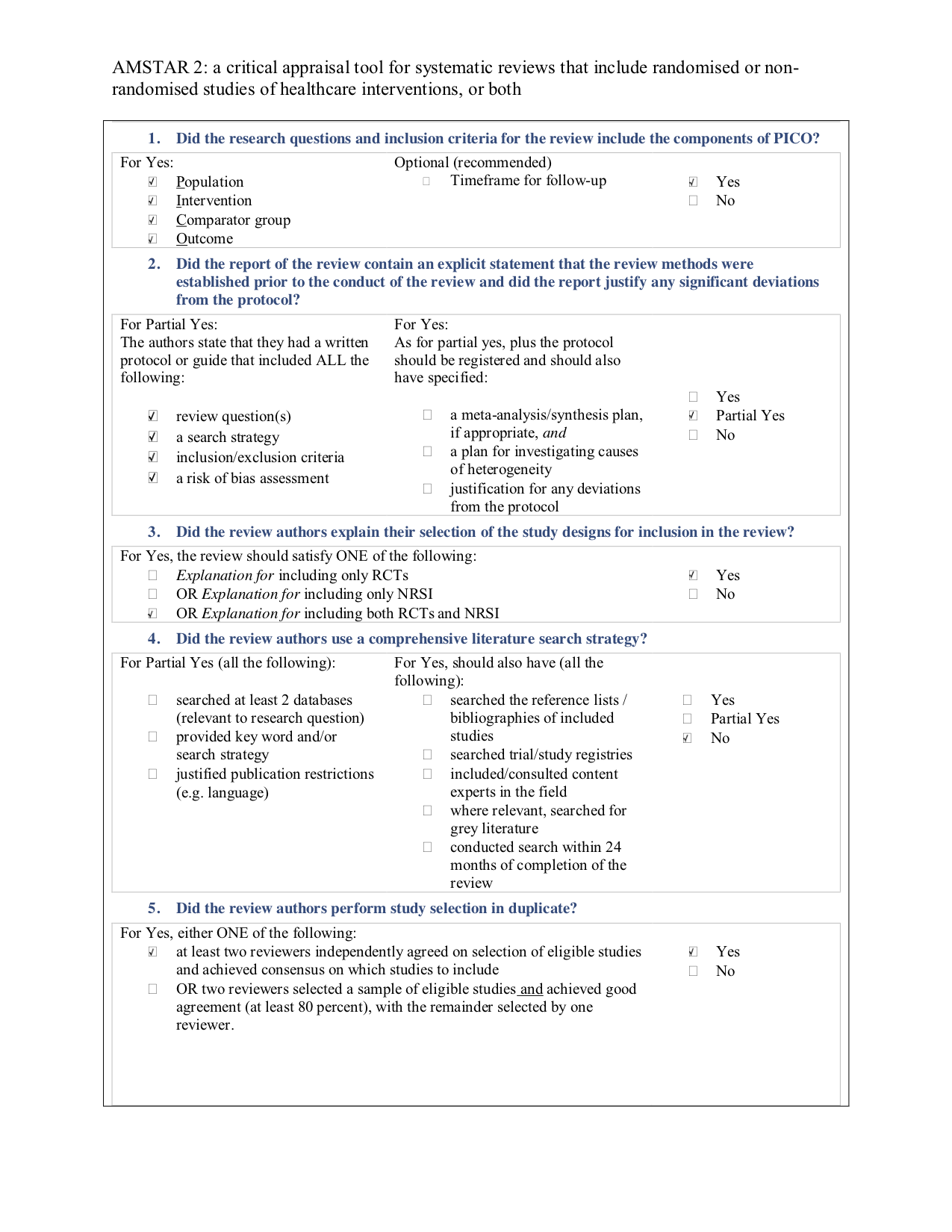


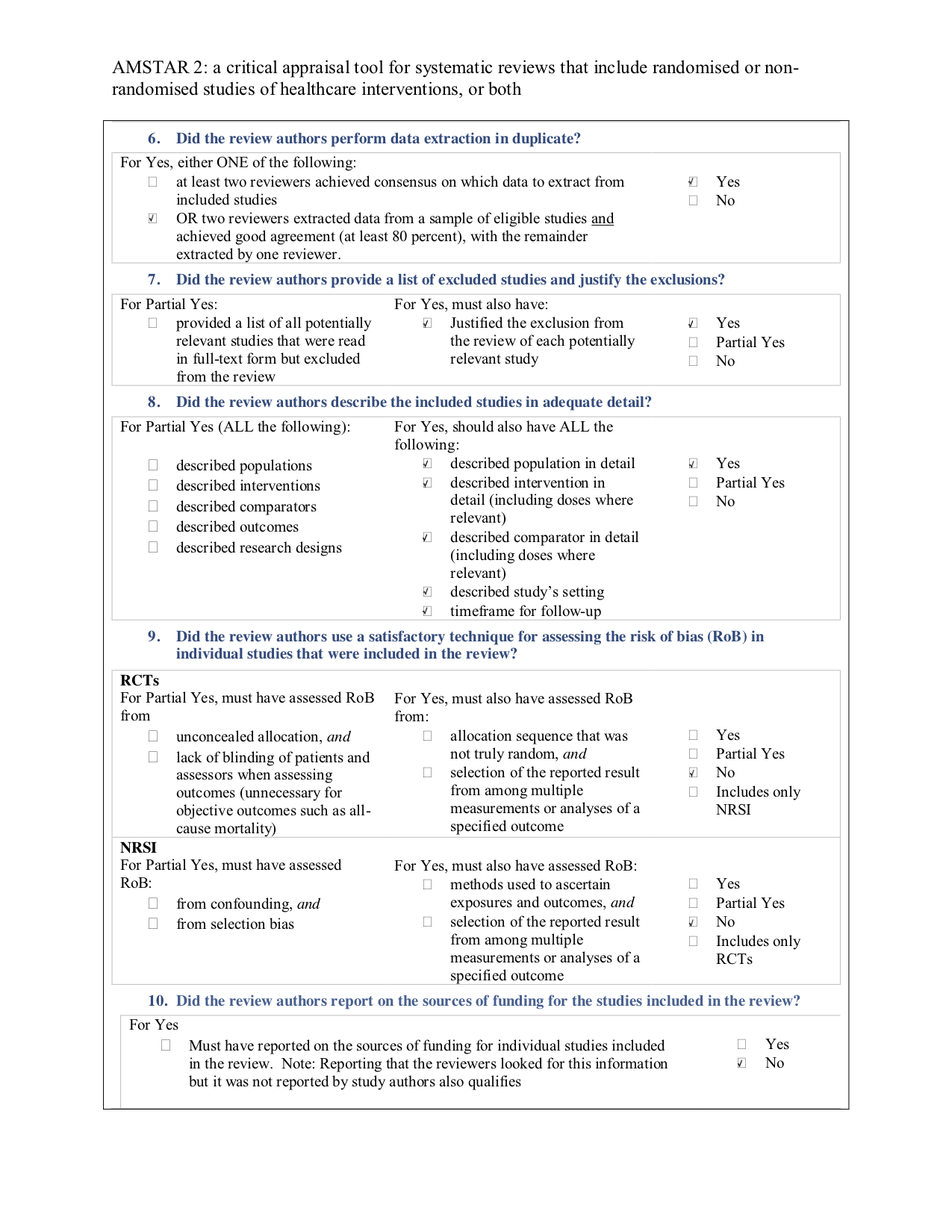


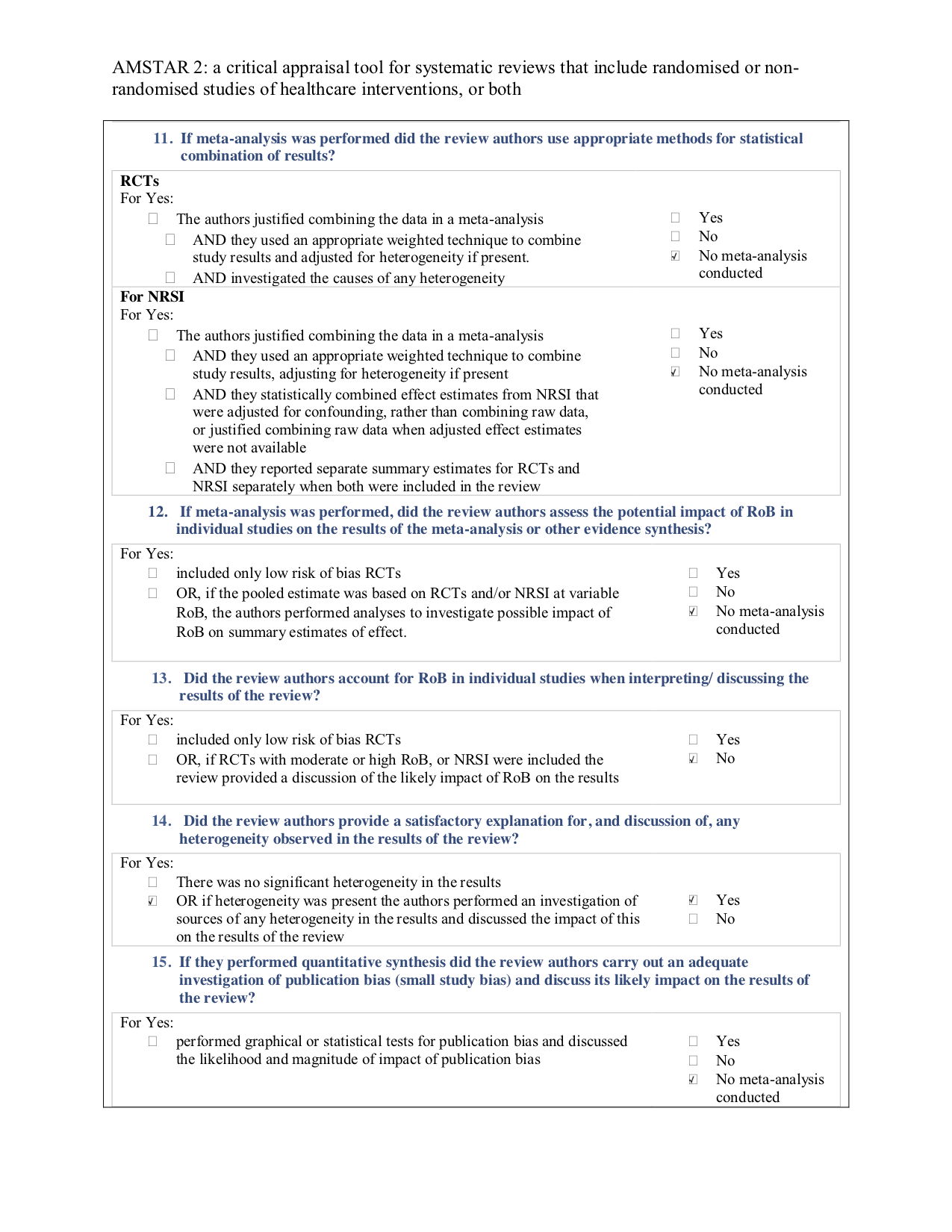


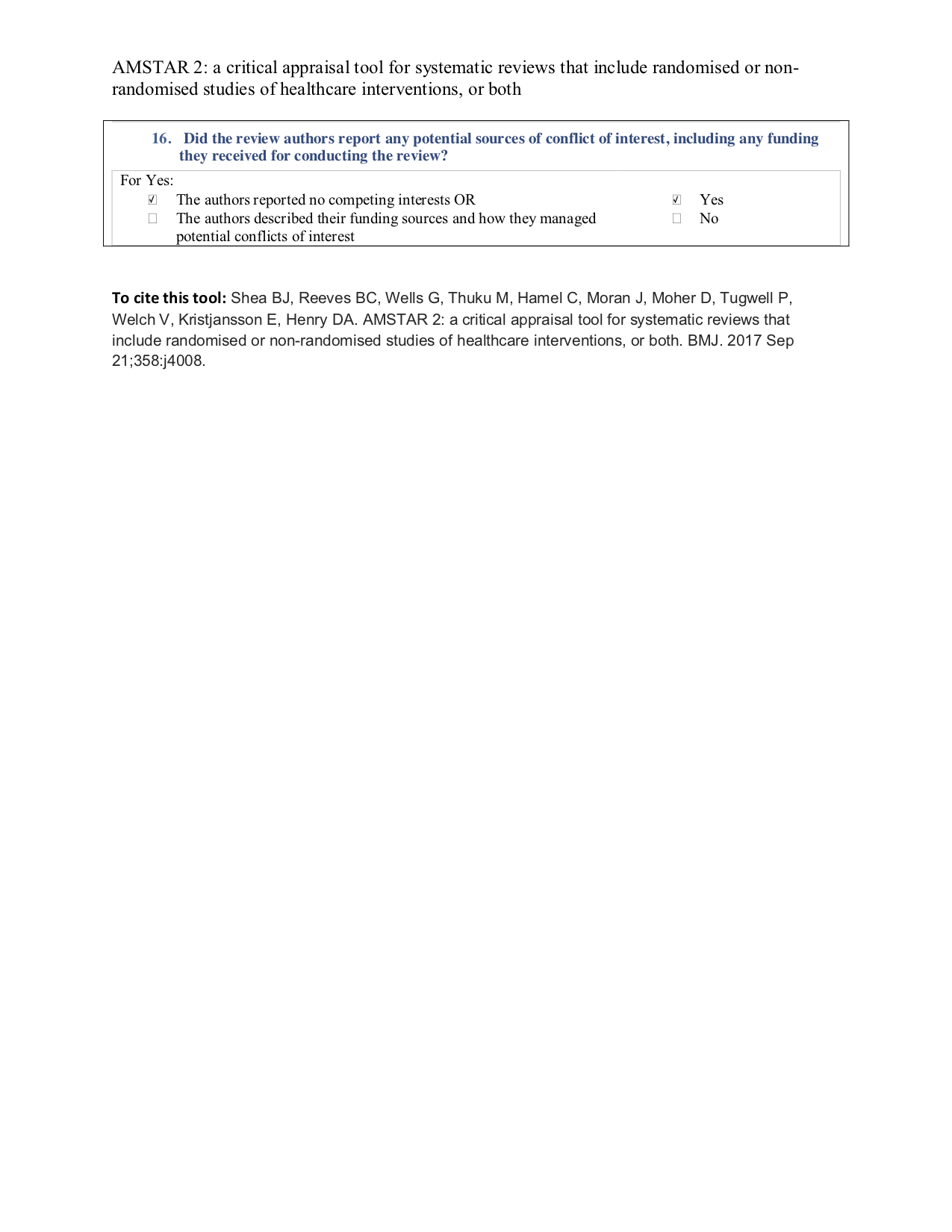

Supplement: AMSTAR 2 [file mmc2.doc]
